# Microvascular Reactivity and Systemic Vascular Resistance Reflect Inflammatory Burden in Psoriasis

**DOI:** 10.3390/biomedicines14081844

**Published:** 2026-08-17

**Authors:** Vanda Bondare-Ansberga, Peteris Tretjakovs, Simons Svirskis, Antra Jurka, Indra Mikelsone, Edgaras Stankevicius, Leons Blumfelds, Ilona Hartmane

**Affiliations:** 1 Department of Dermatology and Venereology, Riga Stradins University, LV-1007 Riga, Latvia; 2 Department of Human Physiology and Biochemistry, Riga Stradins University, LV-1007 Riga, Latvia; 3 Institute of Microbiology and Virology, Riga Stradins University, LV-1007 Riga, Latvia; ssvirskis@latnet.lv; 4 Institute of Physiology and Pharmacology, Lithuanian University of Health Sciences, LT-44307 Kaunas, Lithuania

**Keywords:** psoriasis, psoriasis area and severity index, cytokines, laser Doppler flowmetry, total peripheral resistance

## Abstract

**Background/Objectives:** Psoriasis is a systemic immune-mediated inflammatory disease associated with endothelial dysfunction and cardiovascular risk. This study evaluated relationships among clinical severity, cytokine activity, systemic vascular resistance, and skin microvascular reactivity in chronic plaque psoriasis before and after therapy. **Methods:** In this prospective longitudinal study, 34 patients with chronic plaque psoriasis and 19 matched controls were assessed. Patients were examined at baseline and after 12 months of clinically indicated systemic therapy. Psoriasis Area and Severity Index (PASI), serum IL-17A, IL-22, TNF-α, IL-12(p40), IL-10, VEGF-A, total peripheral resistance, and laser Doppler flowmetry post-occlusive reactive hyperemia indices were analysed. **Results:** PASI decreased markedly after therapy, from 20.6 ± 7.7 to 4.1 ± 5.3, with excellent discrimination between pre- and post-treatment states. IL-17A, IL-22, and IL-12(p40) decreased significantly, while TNF-α remained associated with disease severity. Total peripheral resistance and delayed psoriatic-skin hyperemia persisted and correlated with inflammatory/angiogenic markers, particularly IL-12(p40) and VEGF-A. **Conclusions:** Long-term therapy improves clinical and cytokine profiles in psoriasis, but vascular dysfunction may persist despite cutaneous improvement. Integrated cytokine–vascular assessment may complement PASI for evaluating systemic inflammatory burden and cardiovascular risk.

## 1. Introduction

Psoriasis is a chronic immune-mediated inflammatory disease affecting approximately 2–3% of the adult population worldwide and is increasingly recognised as a systemic disorder rather than an exclusively cutaneous condition. Beyond its characteristic erythematous, scaly plaques, psoriasis is associated with a broad spectrum of comorbidities, including obesity, metabolic syndrome, endothelial dysfunction, and an elevated risk of cardiovascular disease. These associations underscore the importance of understanding psoriasis as a disease process characterised by complex interactions between immune dysregulation, vascular abnormalities, and systemic inflammation [1,2].

At the immunological level, psoriasis is driven predominantly by aberrant activation of the interleukin-23/T helper-17 (IL-23/Th17) and Th1 pathways. Key effector cytokines such as interleukin-17A (IL-17A), interleukin-22 (IL-22), tumour necrosis factor-α (TNF-α), and interleukin-12/23 subunits play central roles in keratinocyte hyperproliferation, impaired differentiation, leukocyte recruitment, and sustained inflammatory signalling. IL-22, in particular, has been shown to promote epidermal thickening and barrier dysfunction, while IL-17A synergizes with TNF-α to amplify inflammatory cascades within psoriatic lesions [3,4,5,6]. Anti-inflammatory mediators such as interleukin-10 (IL-10) are also involved, reflecting compensatory regulatory responses that may be insufficient to counterbalance chronic immune activation [7].

In addition to cytokine-mediated immune activation, angiogenic factors such as vascular endothelial growth factor-A (VEGF A) contribute to microvascular changes that may reflect broader disturbances in vascular regulation [8,9,10,11].

Endothelial dysfunction is a recognised early event in atherosclerosis and cardiovascular disease, conditions that occur with increased prevalence in patients with psoriasis. However, the relationship between systemic vascular resistance, skin microvascular function, and immunological activity in psoriasis remains incompletely understood. Clarifying these links is essential, given the growing emphasis on cardiovascular risk stratification in psoriatic patients [12,13,14].

Cutaneous microvascular reactivity can be assessed non-invasively using laser Doppler flowmetry combined with post-occlusive reactive hyperemia testing, providing insight into functional vascular responses associated with inflammatory disorders [15].

Although numerous studies have investigated cytokines, vascular biomarkers, or microcirculatory parameters separately, relatively few have examined these systems simultaneously in a longitudinal framework [16,17,18].

Moreover, while biological therapies have revolutionised psoriasis management and achieve marked clinical improvement, it remains unclear to what extent immunological remission parallels normalisation of vascular function. Residual microvascular or hemodynamic abnormalities after apparent clinical control may have important implications for long-term cardiovascular risk [19,20].

Systems-level analytical approaches such as receiver operating characteristic (ROC) analysis, hierarchical clustering, and Bayesian network modelling offer powerful tools to explore complex, multivariate relationships among biological variables. These methods allow identification of key nodes within inflammatory–vascular networks and provide insight into how such networks reorganise following therapeutic intervention. Their application to psoriasis research remains limited but holds considerable promise for identifying composite biomarkers and mechanistic pathways.

Therefore, the present study aimed to investigate the relationships among inflammatory cytokines, systemic vascular resistance, microvascular reactivity, and clinical disease severity in patients with chronic plaque psoriasis before and after therapy. By combining conventional statistical analyses with multivariate and network-based approaches, we aimed to elucidate the coordinated behaviour of inflammatory and vascular systems in psoriasis and to determine how these relationships evolve following effective treatment.

This integrative approach seeks to deepen understanding of psoriasis as a systemic inflammatory disease with persistent vascular involvement and to identify physiological and molecular markers that may complement clinical assessment and inform long-term cardiovascular risk evaluation in psoriatic patients.

## 2. Materials and Methods

### 2.1. Study Subjects

This study was conducted as a prospective observational longitudinal study with paired pre- and post-treatment assessments. A total of 34 patients with clinically confirmed chronic plaque psoriasis and 19 age-, sex-, and body mass index-matched healthy controls were enrolled. Psoriasis diagnosis was established by a dermatologist based on standard clinical criteria. Disease severity was evaluated using the PASI (Table 1).

Patients underwent baseline evaluation prior to initiation of indicated therapy and were reevaluated after 12 months of continuous treatment, allowing assessment of longitudinal changes in immunological, vascular, and microcirculatory parameters within the same individuals. Healthy controls were assessed once.

Exclusion criteria included systemic infections, autoimmune comorbidities, severe renal or hepatic disease, malignancies, and other conditions known to significantly alter inflammatory cytokine profiles, such as diabetes mellitus, uncontrolled arterial hypertension and dyslipidemia requiring lipid-lowering drugs and current smoking. All participants provided written informed consent. The study protocol conformed to the Declaration of Helsinki and was approved by the Medical Ethics Committee of Riga Stradins University (approval No. 6-3/98).

### 2.2. Therapeutic Intervention

Psoriasis patients received clinically indicated systemic therapy according to contemporary treatment guidelines and individual clinical characteristics. Treatment selection was determined by the treating physician based on disease severity, prior treatment response, comorbid conditions, and patient-specific factors. The therapies administered to the patients were methotrexate (n = 7), adalimumab (n = 16), guselkumab (n = 2), risankizumab (n = 3), secukinumab (n = 1), and ustekinumab (n = 5), and these treatment regimens remained unchanged throughout the follow-up period. The study reflects a real-world treatment setting, in which therapy was not standardised across patients. All patients remained on stable therapy for the 12-month observation period. This heterogeneous treatment approach was intentionally retained in order to evaluate integrated immunological and vascular responses under routine clinical conditions. Treatment heterogeneity is acknowledged as a limitation but also enhances the external validity of the findings.

### 2.3. Laboratory Assays

The venous blood samples were collected from the study subjects after their overnight fasting; they were centrifuged and stored at −80 °C. Interleukin-17A (IL-17A), interleukin-22 (IL-22), tumour necrosis factor-α (TNF-α), vascular endothelial growth factor-A (VEGF-A), interleukin-12(p40) (IL-12(p40) and interleukin-10 (IL-10) (HCYTA-60K, Luminex Corporation, Austin, TX, USA) were measured in serum by Luminex MAGPIX^®^ System (xMAP^®^ Technology Merck Millipore, Burlington, MA, USA). Concentrations of lipids, glucose, and other routine blood biomarkers were analysed by standard methods.

### 2.4. Assessment of Endothelial Function

**Figure 1:**
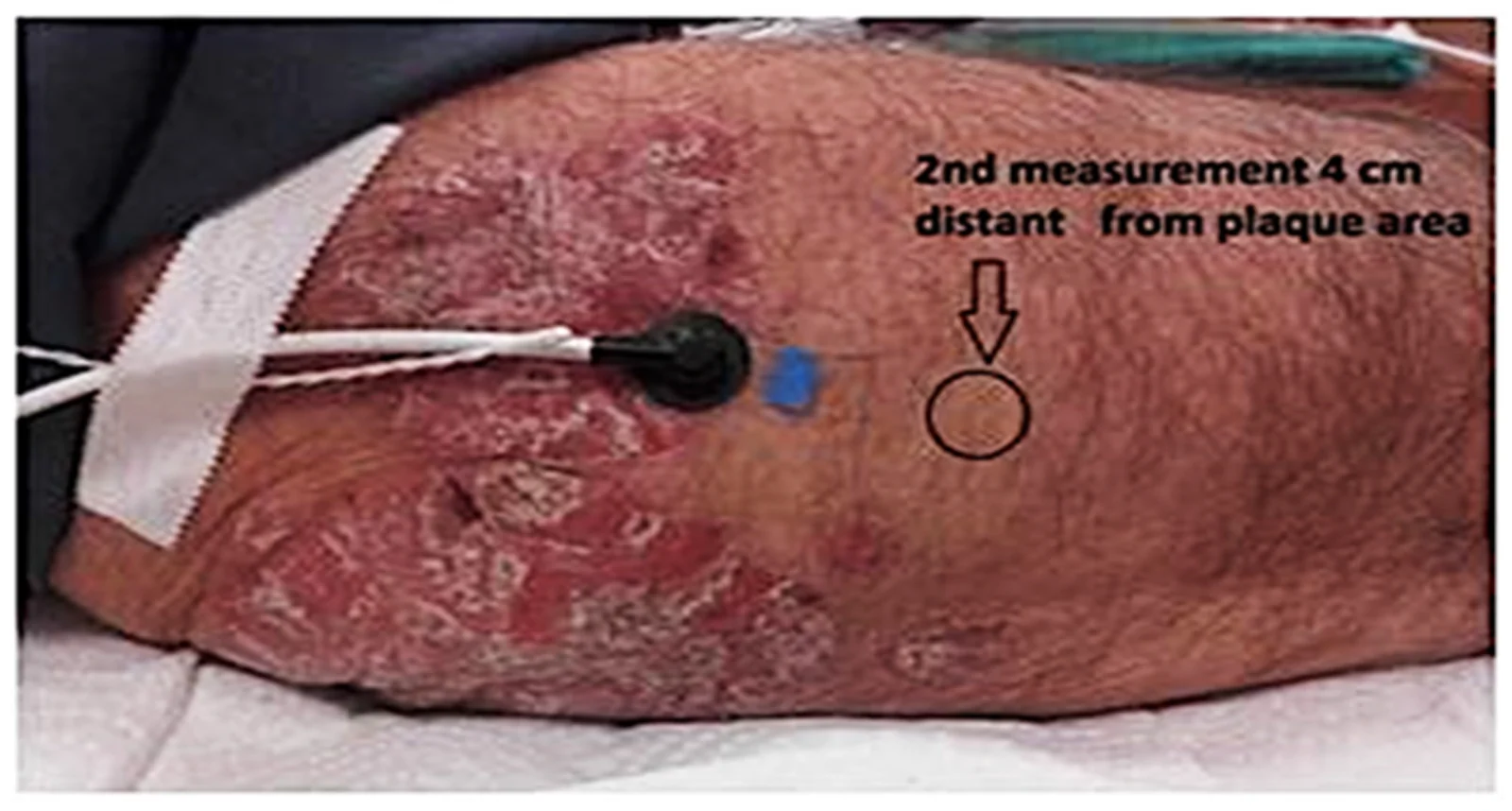
LDF measurement of skin microcirculation.

The skin microvascular endothelial function was evaluated using the LDF technique (Blood Flow Meter INL191, ADInstruments, Oxford, UK). A laser Doppler probe (MSP310XP, ADInstruments, Oxford, UK) was placed on the surface of the psoriasis-affected and adjacent healthy skin of the forearm near the elbow (Figure 1). The probe location was photographed and stored in the study database for repeated blood flow assessment. Microvascular reactivity was evaluated using LDF technology at a laser wavelength of 830 nm in combination with the PORH test. In the PORH test, arterial occlusion was performed with a pressure of 40 mmHg above the systolic arterial pressure by applying a sphygmomanometer cuff on a patient’s upper arm for 2 min. After the release of the pressure, the maximum flux was measured. PORH was registered as perfusion change after 2 min of arterial occlusion. The following microcirculation parameters were determined: resting flow—the flow determined in a resting state before arterial occlusion, biological zero—the flow determined during arterial occlusion, peak flow—the flow determined after release of arterial occlusion. All these parameters are expressed in arbitrary perfusion units [21]. To enhance reproducibility, microvascular reactivity was evaluated by calculating the percentage increase from resting flow. This measure quantifies the magnitude of the vasodilatory response, which is predominantly endothelium-dependent. The time to the hyperemia peak in (1) psoriasis-free skin (Normal hyperemia) and (2) psoriatic skin (Psor hyperemia max) before and after therapy (I and II). The time. This variable is expressed in seconds (s). The time to reach the hyperemia peak provides insight into vascular response kinetics, reflecting the rate at which the microvascular response is generated. The microcirculatory test was performed after a 10–15 min rest with the subjects in a semi-recumbent position in a temperature-controlled room (22–25 °C) in the morning after overnight fasting. Patients were also asked to abstain from smoking, physical exercise, and drinking beverages containing alcohol or caffeine at least 12 h before the study.

To prevent bias, a single investigator, blinded to the group allocation, conducted all tests and data collection. A single investigator conducted all tests and data collection. The LDF device was verified and calibrated using an LDF probe calibration kit in accordance with the manufacturer’s guidelines.

### 2.5. Assessment of Systemic Vascular Resistance

**Figure 2:**
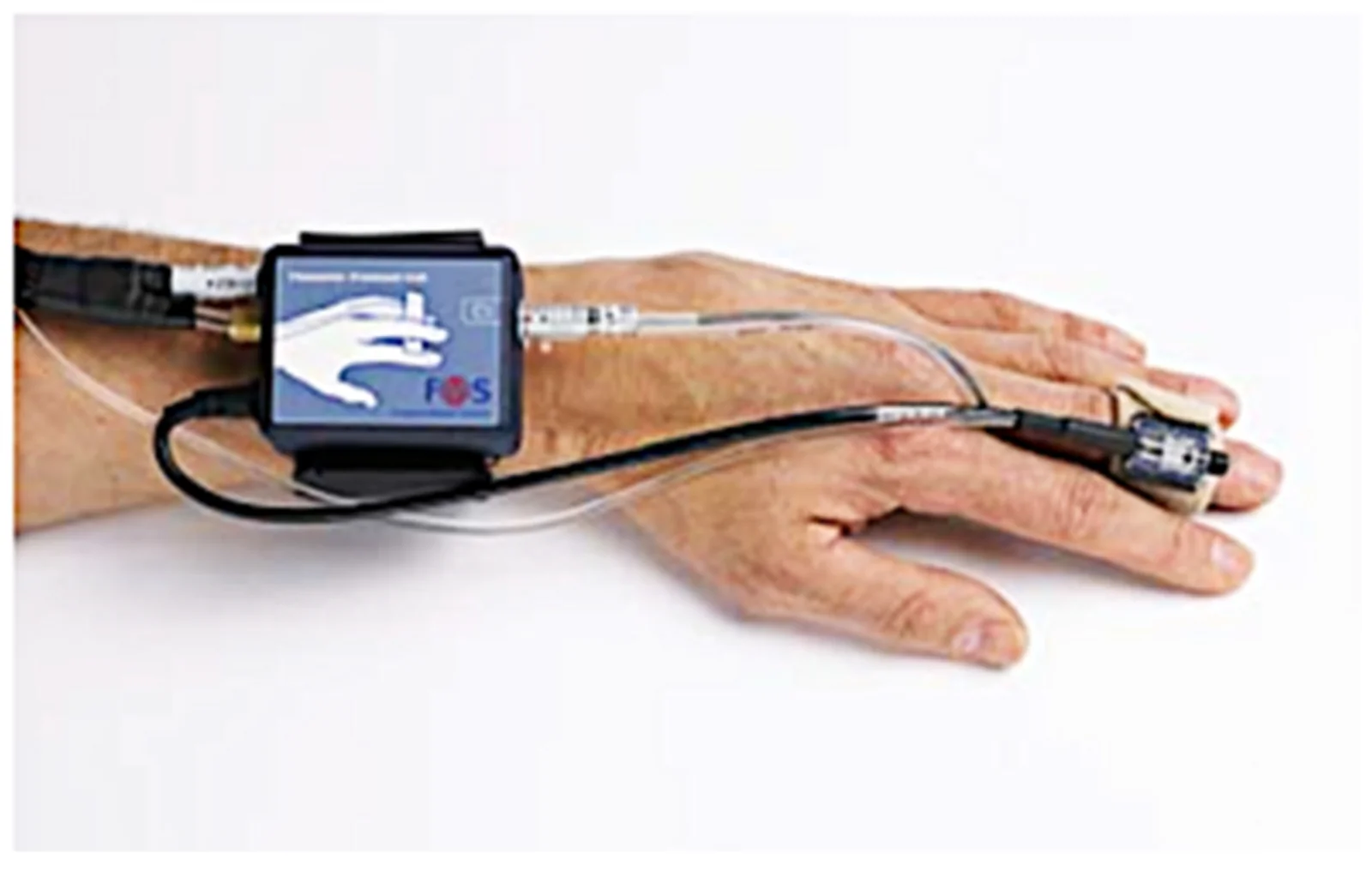
Connecting the finger cuff (Finometer^®^ Model-2 User’s Guide).

Total peripheral resistance (TPR) or systemic vascular resistance is the total resistance the heart must overcome to pump blood through the systemic circulation, calculated as the ratio of mean arterial pressure (MAP) to cardiac output. Data were recorded using the Finometer^®^ Model-2, a non-invasive blood pressure monitoring device (FMS Medical Systems, Amsterdam, The Netherlands), which estimates hemodynamic parameters using finger-cuff technology based on the volume-clamp method (Figure 2) The Hemodynamic BeatScope® Easy software for Finometer® Model-2 uses a statistical model of the human circulation, called ModelFlow, to calculate hemodynamic parameters from the finger arterial pressure waveform, including Stroke Volume (SV), Cardiac Output (CO) and TPR. TPR is determined as the quotient of ModelFlow derived from MAP divided by CO. The unit of measurement for TPR is dyn x s/cm^5^ [22].

### 2.6. Statistical Analysis

Normality of data distribution was assessed using the D’Agostino–Pearson and Shapiro–Wilk tests. Between-group comparisons were performed using unpaired t tests or the Welch t test where appropriate. Non-normally distributed variables were analysed using the Mann–Whitney U test. Paired comparisons before (pre) and after (post) therapy were performed using paired *t* tests or Wilcoxon signed rank tests as appropriate.

To account for multiple comparisons across the six paired cytokine pre-to-post-treatment comparisons, *p*-values were adjusted using the Benjamini–Hochberg false discovery rate (FDR) procedure (Q = 5%). FDR-adjusted q-values are reported alongside uncorrected *p*-values; q < 0.05 was considered statistically significant after correction.

Given the exploratory nature of the study and the evaluation of multiple cytokines and vascular indices, statistical analyses were undertaken with an emphasis on identifying biologically plausible associations and patterns rather than definitive causal inference. Receiver operating characteristic (ROC) curve analyses comparing pre- and post-treatment states were used not as diagnostic classifiers but as standardised indices of biomarker responsiveness to therapy; the AUC in this context quantifies the probability that a post-treatment value is directionally consistent with expected therapeutic change relative to the pre-treatment distribution, providing a complement to paired statistical comparisons.

Correlation analyses were conducted using Spearman’s rank correlation coefficients. Associations with *p* values between 0.05 and 0.10 were interpreted as trend-level, hypothesis-generating findings.

In order to more accurately determine the relationships and conditional dependencies of the variables under study, Bayesian network analysis was used as a probabilistic graphical model.

To uncover latent organisational patterns within the multidimensional dataset, Ward’s minimum variance hierarchical clustering was used, grouping participants according to overall physiological similarity and exposing underlying subgroup structure. However, multivariate analyses were undertaken with the primary objective of identifying biologically coherent structural patterns within the data, and independent replication in larger, prospectively recruited cohorts is a prerequisite for generalisation of the observed network topology.

All analyses were performed using GraphPad Prism (version 9.0, GraphPad Software, San Diego, CA, USA), JMP Pro (version 17; SAS Institute Inc., Cary, NC, USA) and JASP (version 0.95.4; JASP Team, 2024). A two-sided *p* value < 0.05 was considered statistically significant.

## 3. Results

### 3.1. PASI, TPR and Cytokine Changes as a Result of Therapy

**Figure 3:**
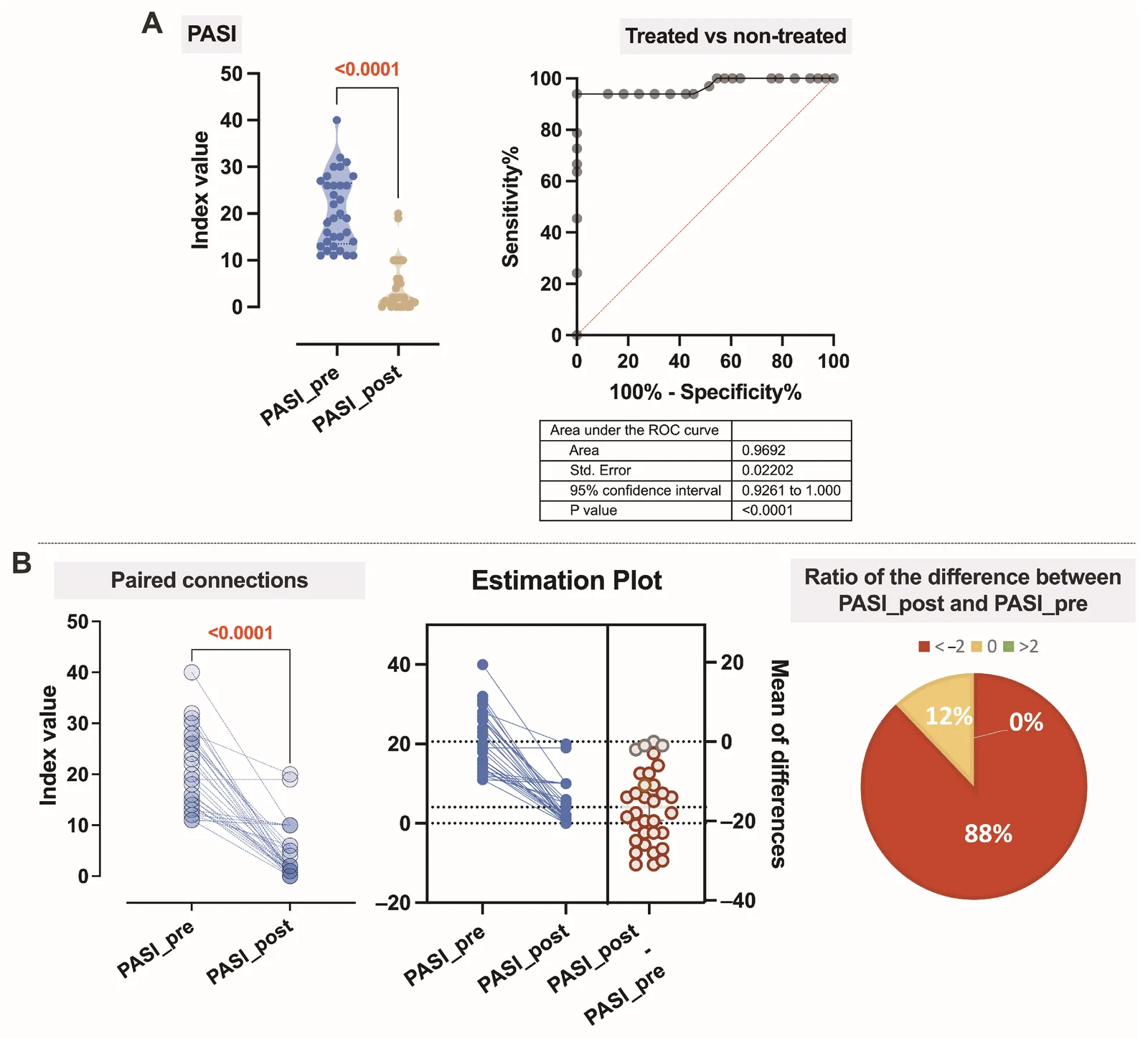
Clinical improvement in psoriasis severity following 12 months of therapy. (**A**) Non-paired comparison and receiver operating characteristic (ROC) curve evaluating the ability of PASI to discriminate between pre- and post-treatment states; (**B**) paired estimation plot showing individual patient trajectories and the mean difference between PASI before (PASI_pre) and after therapy (PASI_post). Yellow circles indicate patients without significant changes in PASI (>−2–<2), red circles indicate those with decreases in PASI (<−2).

Therapy produced a marked reduction in disease severity (Figure 3). PASI scores decreased significantly from baseline to 12 months, with the paired estimation plot showing that 88% of patients experienced improvement, while only 12% showed minimal or no change. The mean difference between PASI before therapy (PASI_pre) and PASI after therapy (PASI_post) was highly significant (*p* < 0.0001). The ROC analysis confirmed that PASI strongly discriminated between treated and untreated states, with an AUC of 0.9692 (95% CI 0.9261–1.000, *p* < 0.0001).

**Figure 4:**
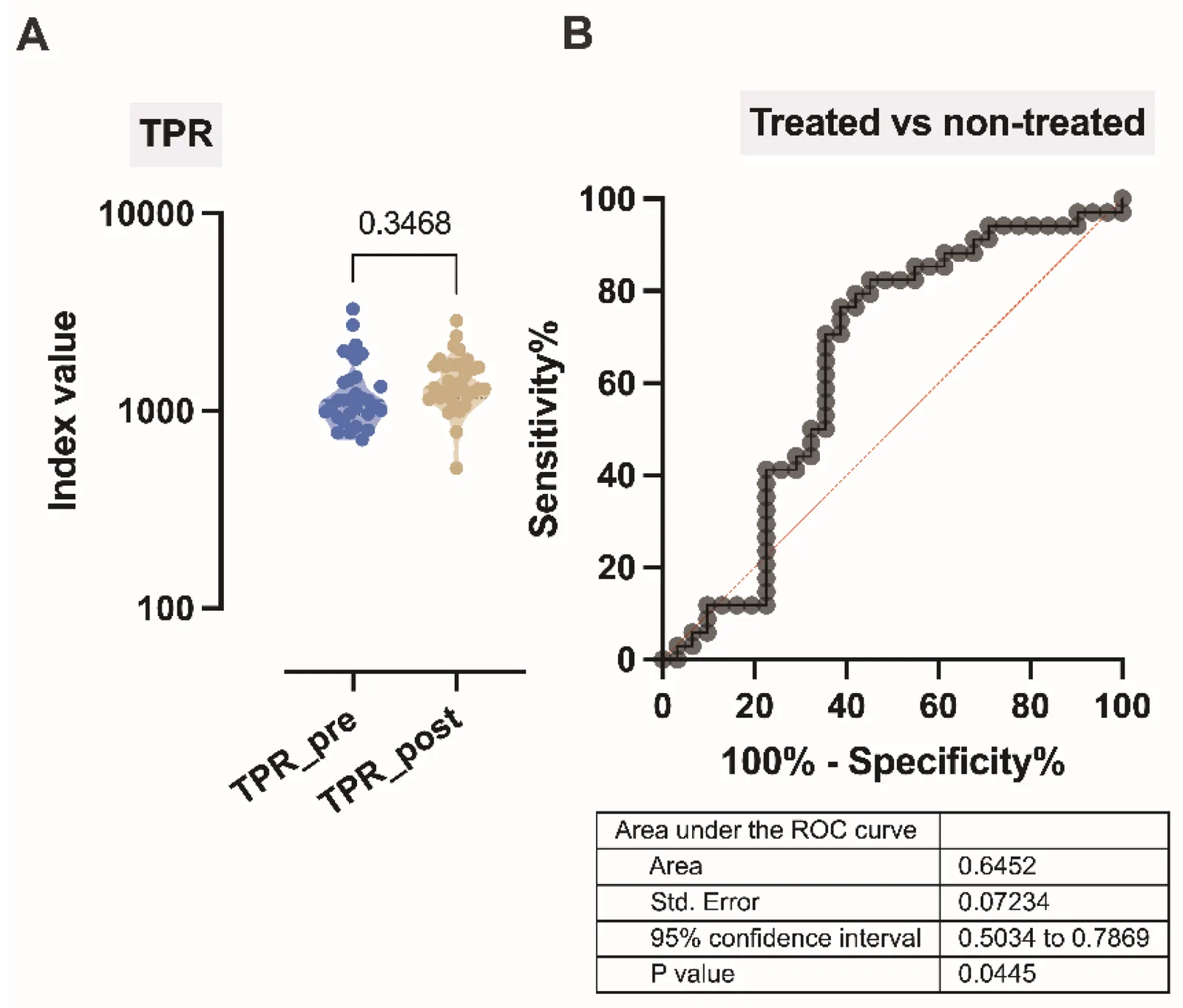
Total peripheral resistance (TPR) before (TPR_pre) and after therapy (TPR_post) (**A**). ROC curve assessing TPR as a discriminator of treatment status (**B**).

TPR demonstrated a statistically insignificant change following therapy (*p* = 0.3468), but the ROC curve yielded an AUC of 0.6452 (*p* = 0.0445), indicating modest classification ability (Figure 4).

**Figure 5:**
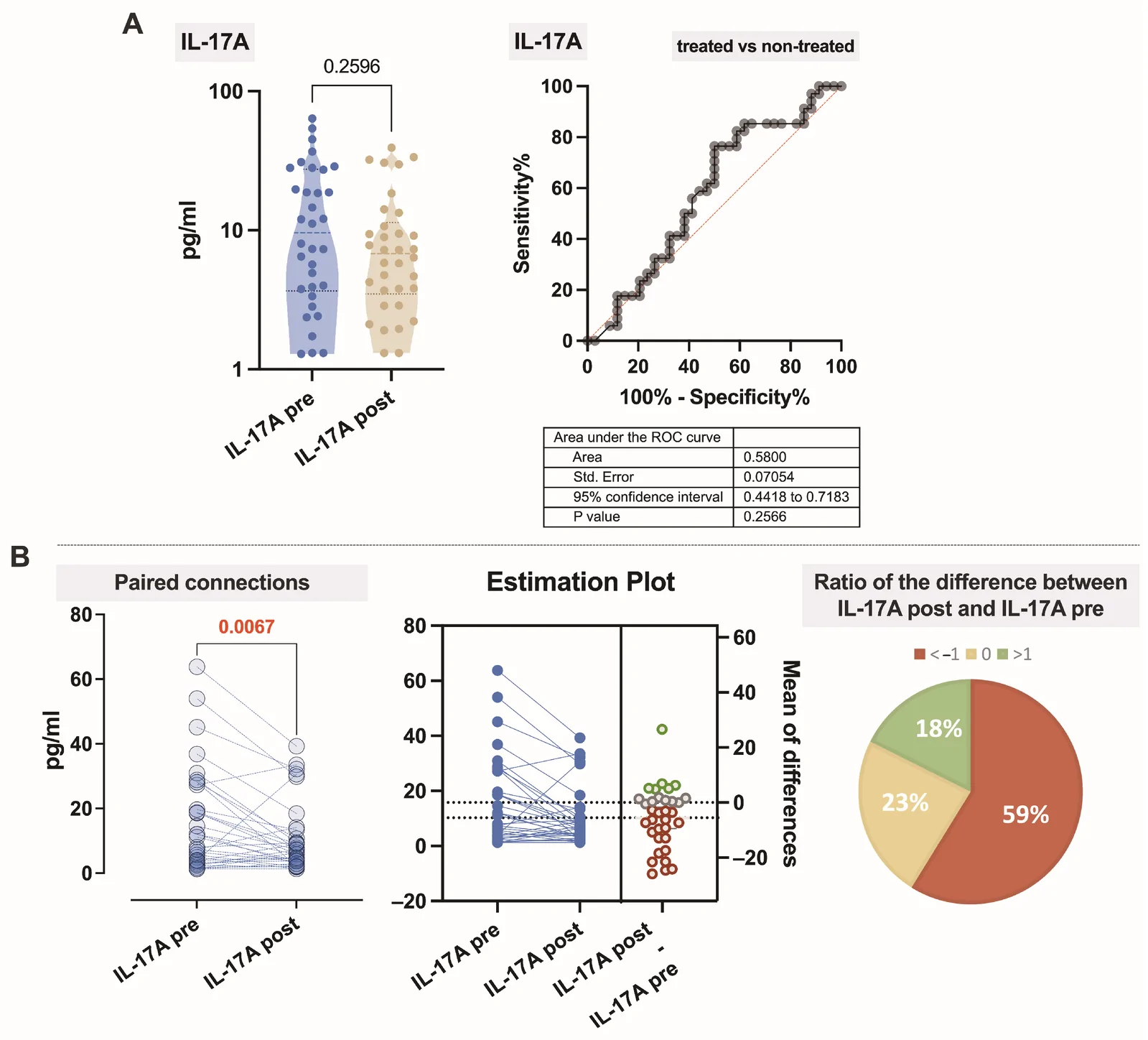
Serum IL-17A concentrations before (IL-17A_pre) and after therapy (IL-17A_post). (**A**) Non-paired comparison and ROC curve before therapy and after therapy; (**B**) paired estimation that shows significant decrease in IL-17A after therapy. Green circles indicate patients with increased IL-17A levels (>1), yellow circles indicate patients without significant changes (>−1–<1), red circles indicate those with decreased IL-17A levels (<1).

IL-17A levels before therapy (IL-17A_pre) decreased significantly after therapy (IL-17A_post) (*p* = 0.0067), although the ROC curve did not reach significance (AUC 0.5800, *p* = 0.2566) (Figure 5). This indicates that IL-17A responds to therapy but is not a strong classifier of treatment status.

**Figure 6:**
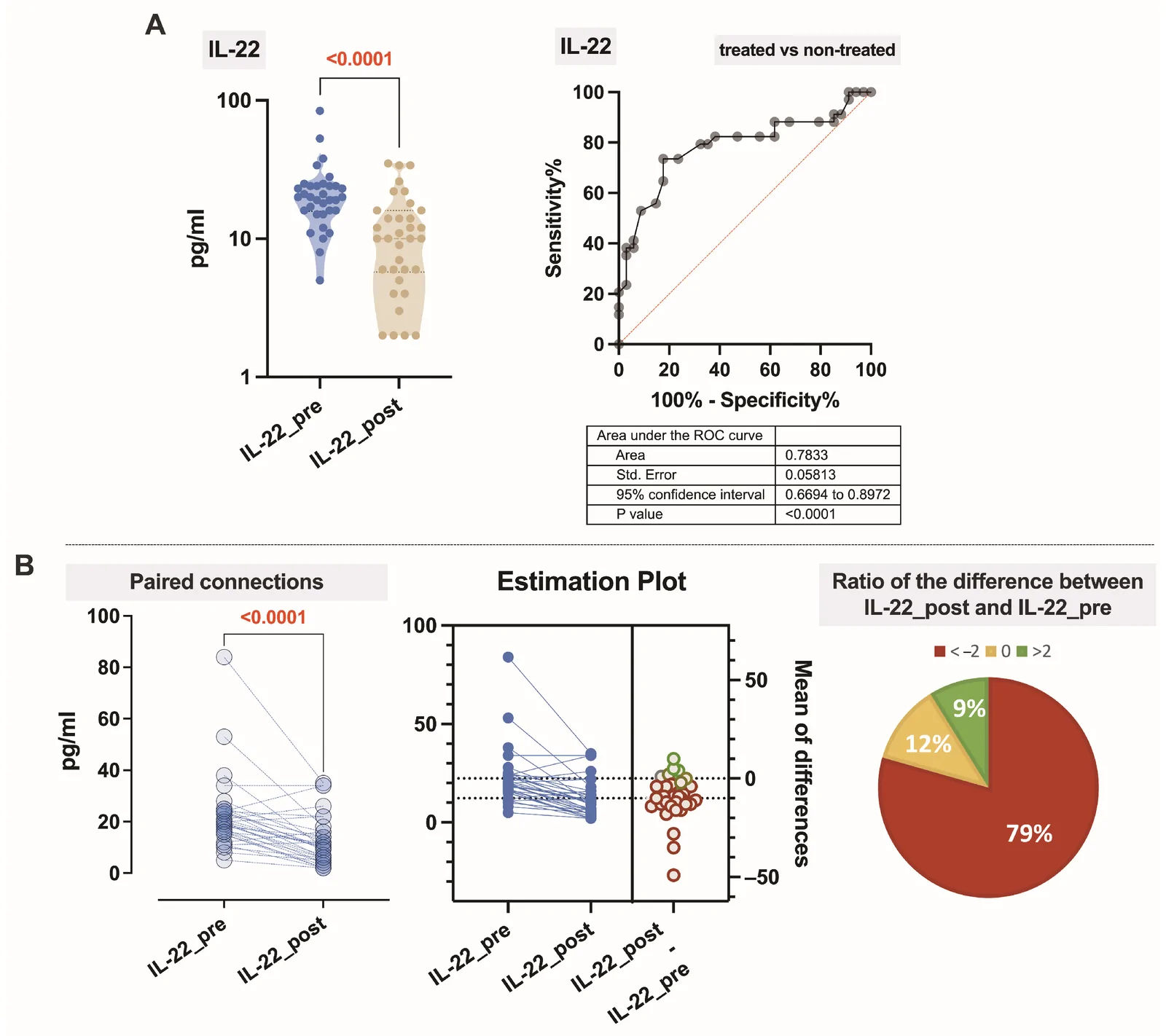
Serum IL-22 concentrations before (IL-22_pre) and after therapy (IL-22_post). (**A**) Non-paired comparison and ROC curve before therapy and after therapy; (**B**) paired estimation that shows significant decrease in IL-22 after therapy. Green circles indicate patients with increased IL-22 levels (>2), yellow circles indicate patients without significant changes (>−2–<2), red circles indicate those with decreased IL-22 levels (<2).

IL-22 showed one of the most pronounced treatment-related reductions—IL-22 levels before therapy (IL-22_pre) decreased significantly after therapy (IL-22_post). The ROC curve demonstrated good discriminatory ability (AUC 0.7833, *p* < 0.0001), and paired analyses confirmed a highly significant decline (*p* < 0.0001) with 79% of patients showing improvement (Figure 6).

**Figure 7:**
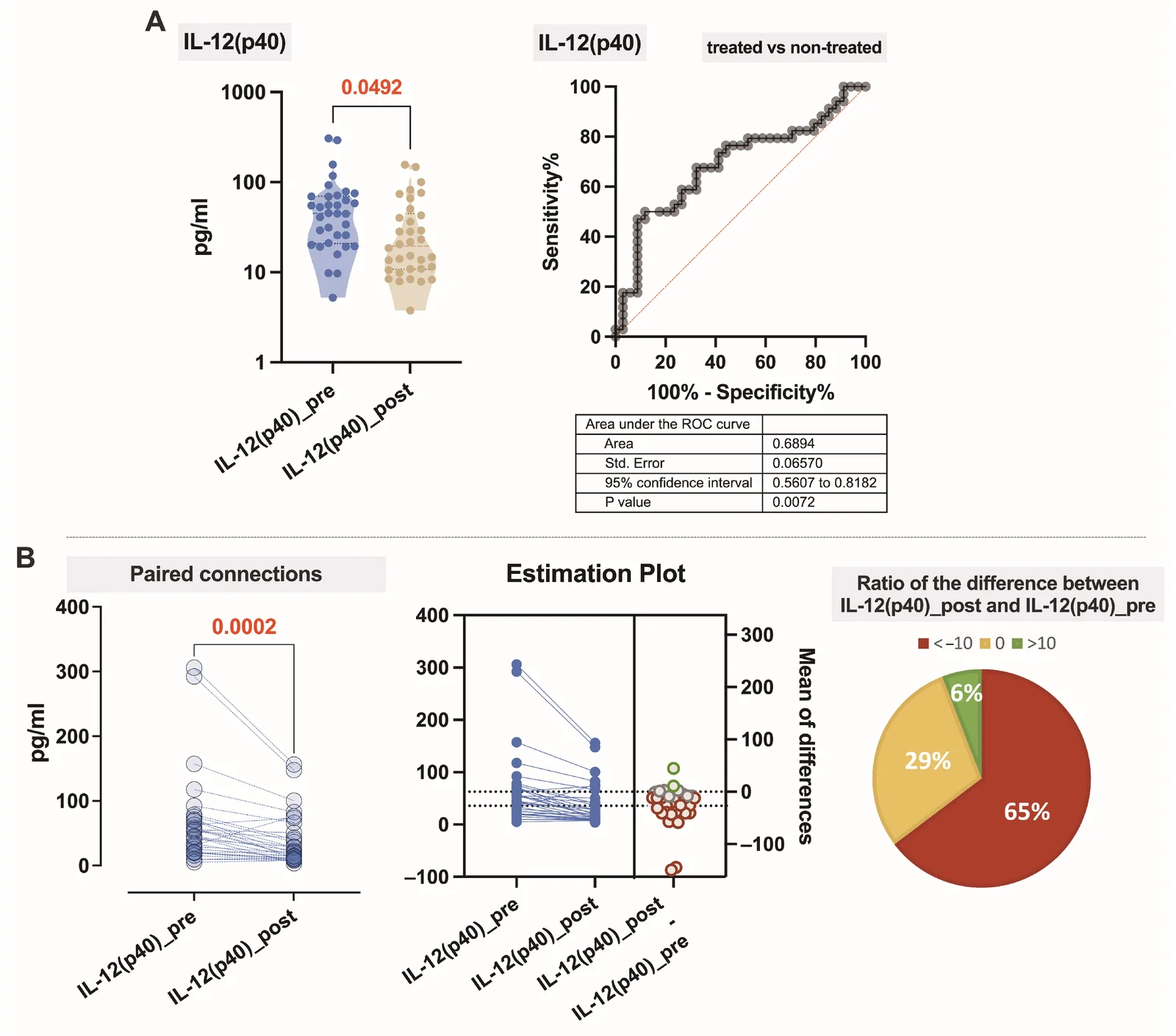
Serum IL-12(p40) concentrations before (IL-12(p40) _pre) and after therapy (IL-12(p40) _post). (**A**) Non-paired comparison and ROC curve before therapy and after therapy; (**B**) paired estimation that shows significant decrease in IL-12(p40) after therapy. Green circles indicate patients with increased IL-17A levels (>10), yellow circles indicate patients without significant changes (>−10–<10), red circles indicate those with decreased IL-12(p40) levels (<10).

IL-12(p40) levels decreased significantly after therapy—IL-12(p40) levels before therapy (IL-12(p40)_pre) decreased significantly after therapy (IL-12(p40) _post) (*p* = 0.0002). The ROC curve indicated moderate discriminatory value (AUC 0.6894, *p* = 0.0072) (Figure 7).

Among the biomarkers examined, PASI (AUC = 0.9692) and IL-22 (AUC = 0.7833) demonstrated high responsiveness to therapy, IL-12(p40) (AUC = 0.6894) and TPR (AUC = 0.6452) showed moderate responsiveness, and IL-17A (AUC = 0.5800, *p* = 0.2566) did not reach significance, indicating that despite a statistically significant paired reduction (*p* = 0.0067, q = 0.0132 after FDR correction), IL-17A levels were not consistently directionally lower post-treatment across the cohort, likely reflecting inter-individual variability in therapeutic suppression.

After FDR correction, four of the six cytokines reached statistical significance as treatment-responsive discoveries: IL-22 (q < 0.001), IL-12(p40) (q = 0.0006), IL-17A (q = 0.0132), and VEGF-A (q = 0.0132). TNF-α (*p* = 0.0801, q = 0.0961) and IL-10 (*p* = 0.1023, q = 0.1023) did not reach FDR-corrected significance and are interpreted as trend-level findings. The paired *p*-values and FDR-adjusted q-values for all six cytokines are presented in Table 2.

### 3.2. Correlations Between Cytokine Levels and Also with PASI

**Figure 8:**
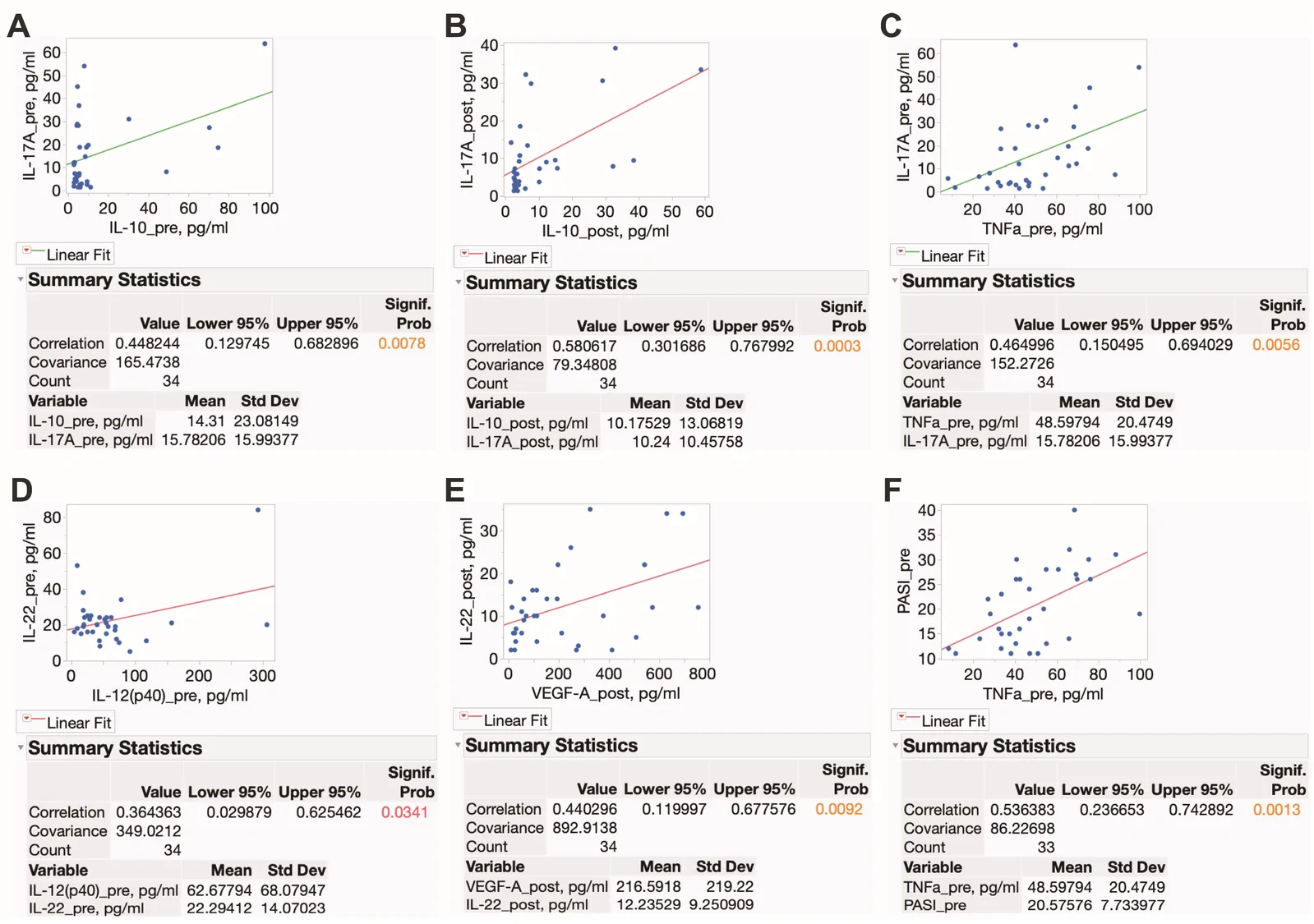
Multiple correlation analyses showing significant associations between inflammatory cytokines as well as with PASI. Figure shows correlations: IL-17A before (IL-17A_pre, pg/mL) and after therapy (IL-17A_post, pg/mL) and IL-10 before (IL-10_pre, pg/mL) and after therapy (IL-10_post, pg/mL) (**A**,**B**); IL-17A pg/mL and TNF-a before therapy (IL-17A_pre, pg/mL and TNF-a_pre, pg/mL) (**C**). IL 22 before (IL-22_pre, pg/mL) and after therapy (IL-22_post, pg/mL), and IL-12(p40) before therapy (IL-12(p40)_pre, pg/mL) (**D**) and with VEGF-A after therapy (VEGF-A_post, pg/mL) (**E**). TNF α before therapy (TNF α_pre, pg/mL) and PASI before therapy (PASI_pre) (**F**).

IL-10 correlated positively with IL-7A before and after therapy (r = 0.4482, *p* = 0.0078 and r = 0.5806, *p* = 0.0003; Figure 8A,B); moreover, IL-17A correlated positively with TNF-a before therapy (Figure 8C), suggesting a coordinated regulatory response within the cytokine network. TNF-α remained strongly associated with disease severity. IL-22 correlated positively with IL-12(p40) before therapy and with VEGF-A after therapy (r = 0.3643, *p* = 0.0341 and r = 0.4402, *p* = 0.0092; Figure 8D,E), indicating a link between inflammatory and angiogenic pathways in the post-treatment state. A significant positive correlation was observed between TNF-α and PASI before therapy (r = 0.536, *p* = 0.0013; Figure 8F).

### 3.3. Relationship Between TPR and Studied Cytokines

**Figure 9:**
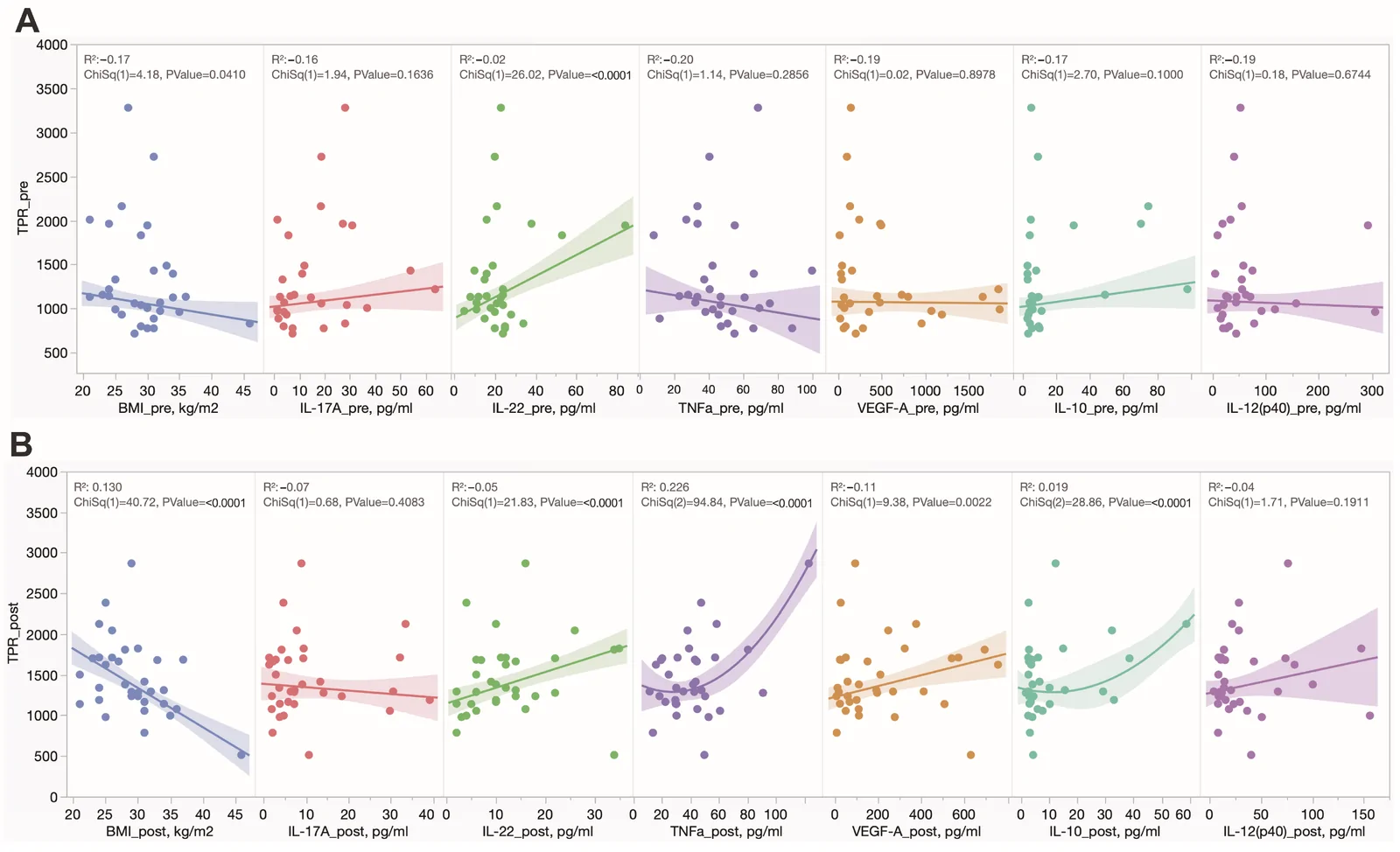
Relationship between TPR and cytokines ((**A**)—before treatment, (**B**)—after treatment). Chi-square analyses evaluating the relationship between systemic vascular resistance (TPR) and cytokines. TNFa demonstrated the strongest association with TPR (χ^2^ = 94.84, *p* < 0.0001), while other cytokines showed weaker or nonsignificant relationships.

The associations between total peripheral resistance (TPR) and BMI plus all six cytokines were evaluated by chi-square analysis with fitted regression lines and 95% confidence bands, presented separately for the pre-treatment state (Panel A) and post-treatment state (Panel B) in Figure 9.

Panel A—Pre-treatment. Of the seven variables assessed against pre-treatment TPR, two reached statistical significance. IL-22 demonstrated the strongest and most visually evident positive association (χ^2^(1) = 26.02, *p* < 0.0001): the regression line shows a clear positive slope with a moderately narrow confidence band, indicating that higher baseline IL-22 concentrations were associated with higher systemic vascular resistance. BMI showed a statistically significant but modest negative association with pre-treatment TPR (χ^2^(1) = 4.18, *p* = 0.0410), with higher BMI values corresponding to slightly lower TPR. The remaining five variables—IL-17A (χ^2^(1) = 1.94, *p* = 0.1636), TNF-α (χ^2^(1) = 1.14, *p* = 0.2856), VEGF-A (χ^2^(1) = 0.02, *p* = 0.8978), IL-10 (χ^2^(1) = 2.70, *p* = 0.1000), and IL-12(p40) (χ^2^(1) = 0.18, *p* = 0.6744)—did not demonstrate statistically significant associations with pre-treatment TPR, with correspondingly wide confidence bands and flat or near-horizontal fitted lines across all five scatterplots.

Panel B—Post-treatment. Following therapy, the pattern of TPR associations changed substantially, with five of the seven variables reaching statistical significance. BMI emerged as the strongest overall post-treatment TPR correlate (R^2^ = 0.130, χ^2^(1) = 40.72, *p* < 0.0001), displaying a pronounced negative linear relationship with a narrow confidence band—the most visually well-defined association in the entire figure. TNF-α demonstrated the most striking post-treatment association (R^2^ = 0.226, χ^2^(2) = 94.84, *p* < 0.0001), characterised by a steep exponential-like fitted curve: TPR values remained relatively stable at low-to-moderate TNF-α concentrations and then increased sharply at the highest TNF-α values, with the curve extending upward toward approximately 3000 dyn·s/cm^5^ at the upper end of the TNF-α distribution. This non-linear profile, reflected in the two-degree-of-freedom chi-square statistic, indicates that the relationship between TNF-α and vascular resistance is not adequately captured by a linear model and may reflect a threshold or accelerating effect at high inflammatory burden. IL-22 retained a significant positive association with post-treatment TPR (χ^2^(1) = 21.83, *p* < 0.0001), with a clear upward-sloping regression line and moderate confidence band width. VEGF-A demonstrated a significant positive association (χ^2^(1) = 9.38, *p* = 0.0022), with a moderate positive slope and broader confidence band reflecting greater variability. IL-10 showed a significant non-linear positive association (χ^2^(2) = 28.86, *p* < 0.0001), with an exponential-like fitted curve indicating accelerating TPR at higher IL-10 concentrations. In contrast, IL-17A (χ^2^(1) = 0.68, *p* = 0.4083) and IL-12(p40) (χ^2^(1) = 1.71, *p* = 0.1911) did not reach statistical significance post-treatment, with flat fitted lines and wide confidence bands.

Taken together, these findings reveal a marked change in the cytokine–vascular resistance landscape following therapy. Pre-treatment TPR was significantly associated only with IL-22 and BMI, while post-treatment TPR showed significant associations with five variables: BMI (negative, linear), TNF-α (positive, non-linear), IL-22 (positive, linear), VEGF-A (positive, linear), and IL-10 (positive, non-linear). The emergence of BMI as the dominant post-treatment TPR correlate and the pronounced exponential TNF-α–TPR relationship after therapy suggest that residual vascular resistance following clinical improvement is driven by a combination of metabolic burden and persistent low-grade pro-inflammatory signalling rather than the angiogenic (IL-22) pathway that dominated pre-treatment associations.

### 3.4. Correlations of Study Markers with LDF PORH Test Indices

**Figure 10:**
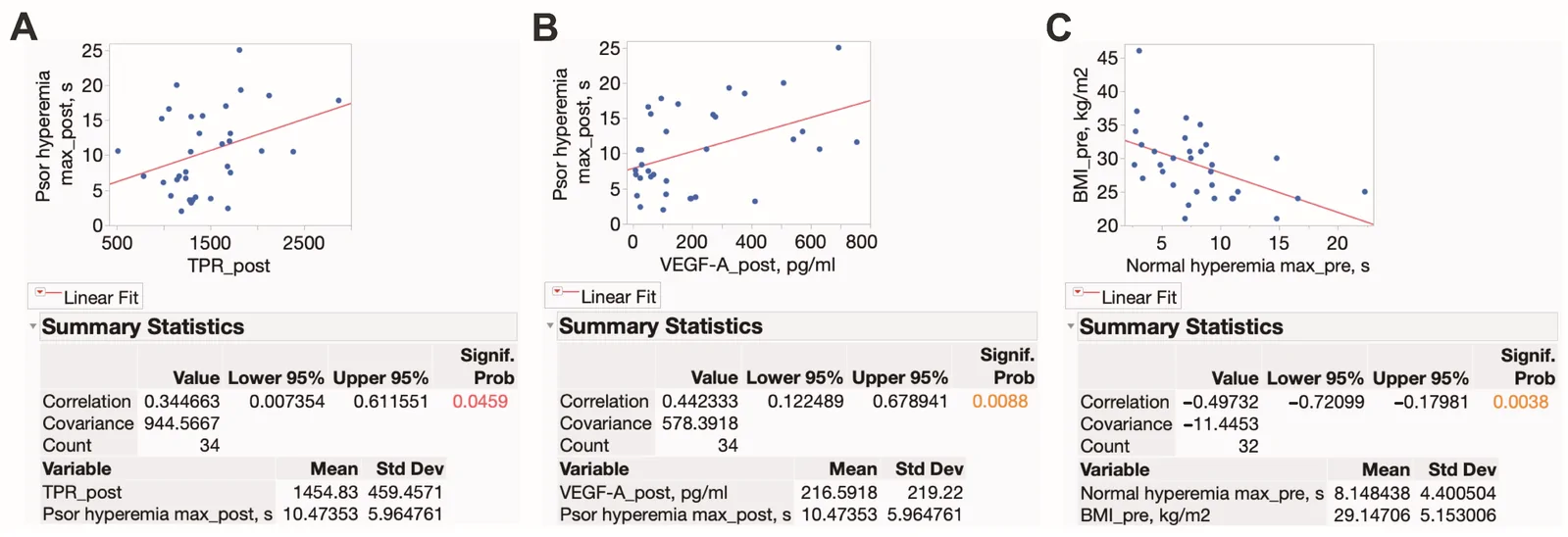
Relationship between: (**A**)—TPR after therapy (TPR_post) and time to peak hyperemia after therapy (Psor hyperemia max_post, s); (**B**)—VEGF-A after therapy (VEGF-A pg/ml_post) and time to peak hyperemia after therapy (Psor hyperemia max_post, s); (**C**)—time to peak hyperemia in psoriasis-free skin before therapy (Psor hyperemia max_pre, s) and BMI_pre.

The time to the hyperemia peak in (1) psoriasis-free skin (Normal hyperemia) and (2) psoriatic skin (Psor hyperemia max) before and after therapy (pre and post). Scatterplot showing the association between total peripheral resistance after therapy (TPR_post) and time to the hyperemia peak in psoriatic skin after therapy (Psor hyperemia max_post, s)—a positive correlation was observed (r = 0.3447, *p* = 0.0459) (Figure 10). VEGF-A after therapy (VEGF-A_post, pg/mL) may indicate significant associations with microvascular indices, particularly psoriatic hyperemia (Psor hyperemia max II, s) (r = 0.442, *p* = 0.0088). Interestingly, body mass index (BMI_pre) correlated with time to the hyperemia peak in psoriasis-free skin before therapy (Normal hyperemia max_pre, s).

### 3.5. Bayesian Network Modelling

**Figure 11:**
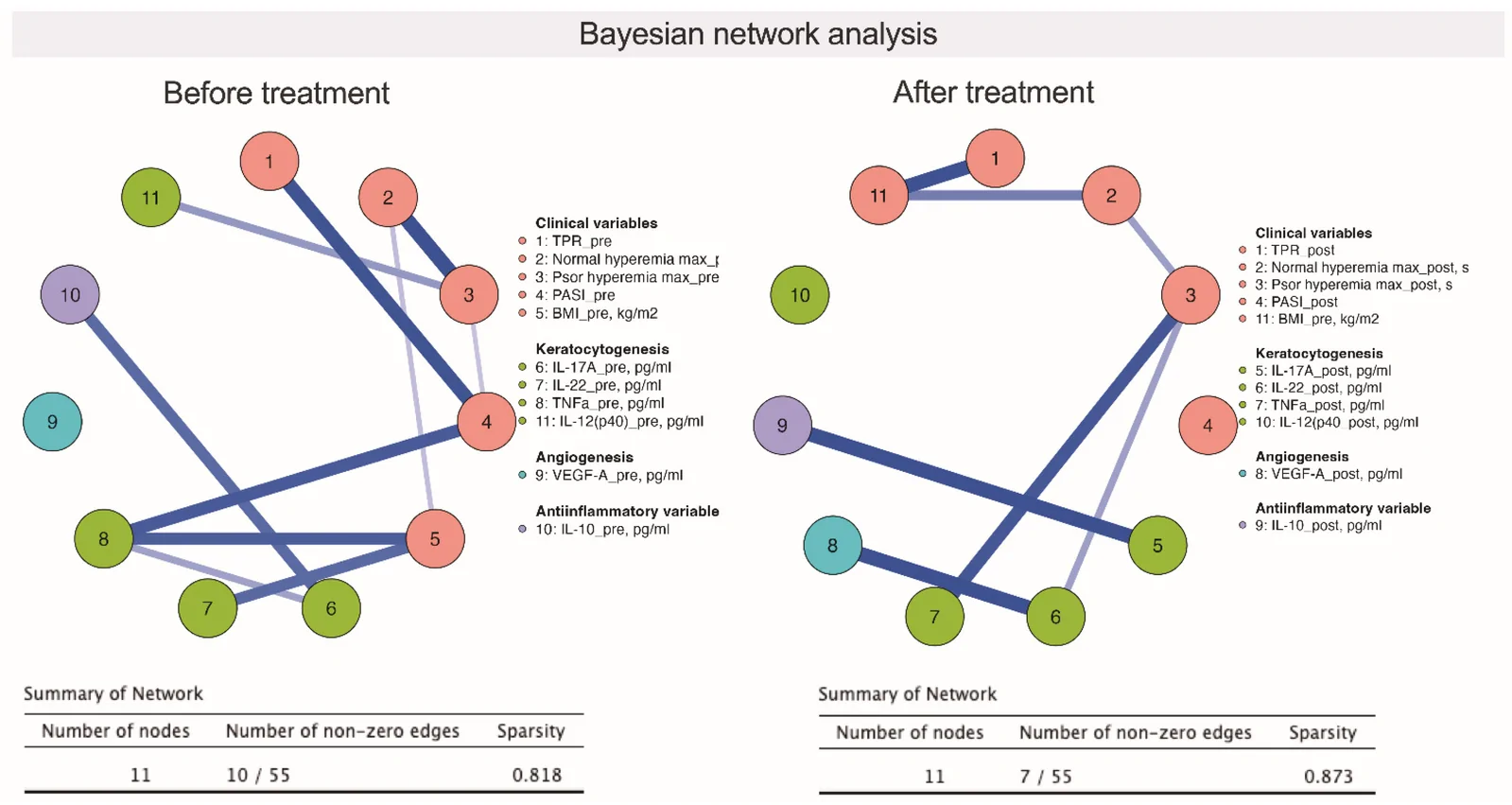
Bayesian network analysis of inflammatory, clinical, and vascular variables. The inflammatory and microvascular system in psoriasis is highly interconnected, with therapy altering network structure showing association change between studied factors.

Bayesian network analysis was applied to the full variable set at both timepoints as an exploratory, hypothesis-generating approach to characterising conditional dependency structure among clinical, immunological, and hemodynamic variables. Given the patient-to-variable ratio of approximately 3:1 (34 patients, 11 variables), all findings reported below should be interpreted as candidate structural patterns rather than confirmed biological mechanisms, and are presented with full quantitative transparency to facilitate independent critical appraisal. Results of Bayesian network analysis of studied variables before and after treatment are represented in Figure 11.

Both networks comprised 11 nodes representing the same variables grouped into four functional categories: clinical variables (TPR, Normal hyperemia max, Psor hyperemia max, PASI, BMI), keratocytogenesis cytokines (IL-17A, IL-22, TNF-α, IL-12(p40)), angiogenesis (VEGF-A), the anti-inflammatory variable (IL-10), and the angiogenesis variable (VEGF-A). The node numbering differs slightly between panels due to renumbering of BMI (node 5 pre → node 11 post) and IL-12(p40) (node 11 pre → node 10 post). The pre-treatment network contained 10 active conditional dependencies out of 55 possible pairwise connections (graph sparsity coefficient = 0.818), indicating a moderately sparse but structured dependency graph. The variable most extensively connected within this network was PASI, which shared active edges with six other variables: TPR, Normal hyperemia max, Psor hyperemia max, BMI, IL-22, and IL-10. This pattern is consistent with the hypothesis that, in the pre-treatment state, clinical disease severity may constitute a shared correlate of microvascular, hemodynamic, and cytokine variation, though the directionality and causal interpretation of these conditional associations cannot be determined from the present observational network structure. Additional notable conditional dependencies in the pre-treatment network included TNF-α with IL-22 and BMI, IL-10 with BMI, and TPR with IL-12(p40). VEGF-A appeared as an isolated node with no active edges, suggesting conditional independence from all other variables in the pre-treatment network, though whether this reflects a genuine biological compartmentalization or a power-related failure to detect weak conditional associations remains unclear given the sample size.

The post-treatment network was more sparse, with 7 active edges out of 55 possible (sparsity = 0.873), indicating a reduction in the number of statistically supported conditional dependencies following therapy. Key changes in network topology included: a reduction in PASI connectivity (now sharing edges primarily with Psor hyperemia max, IL-17A, and IL-10); preservation of the TNF-α/IL-22 conditional dependency; emergence or strengthening of BMI associations with Normal hyperemia max and TPR; and the appearance of a new IL-10/IL-17A conditional dependency absent in the pre-treatment network. VEGF-A remained relatively peripheral, sharing only a single edge with IL-22 post-treatment.

These findings are consistent with the hypothesis that treatment is associated with a simplification of the conditional dependency structure across inflammatory, vascular, and clinical variables. PASI remained a central hub in both states, reinforcing its role as the key integrating clinical variable. The pre-treatment network was predominantly organised around PASI–cytokine–vascular co-dependencies, while the post-treatment network showed a relative shift toward BMI–vascular coupling and an emergent IL-10/IL-17A conditional dependency—a pattern that, if replicated, would be consistent with a hypothesis of immunomodulatory reconfiguration after treatment, though this interpretation remains speculative at the present sample size. However, the observed reduction in active edges (10 → 7) may reflect statistical power limitations as much as genuine biological network reorganisation, and should not be interpreted as evidence of causal therapeutic suppression of specific network interactions. Replication in independently recruited, larger cohorts using confirmatory network methods is required before the structural features identified here can be considered generalizable.

### 3.6. Hierarchical Clustering with Ward Method

Ward’s minimum variance hierarchical clustering was applied to pre- and post-treatment data as a complementary exploratory approach to identify latent subgroup structure within the cohort. As with the Bayesian network analysis, these results are presented as hypothesis-generating observations in the context of a modest sample size (n = 34) and should not be interpreted as defining biologically validated patient subtypes.

**Figure 12:**
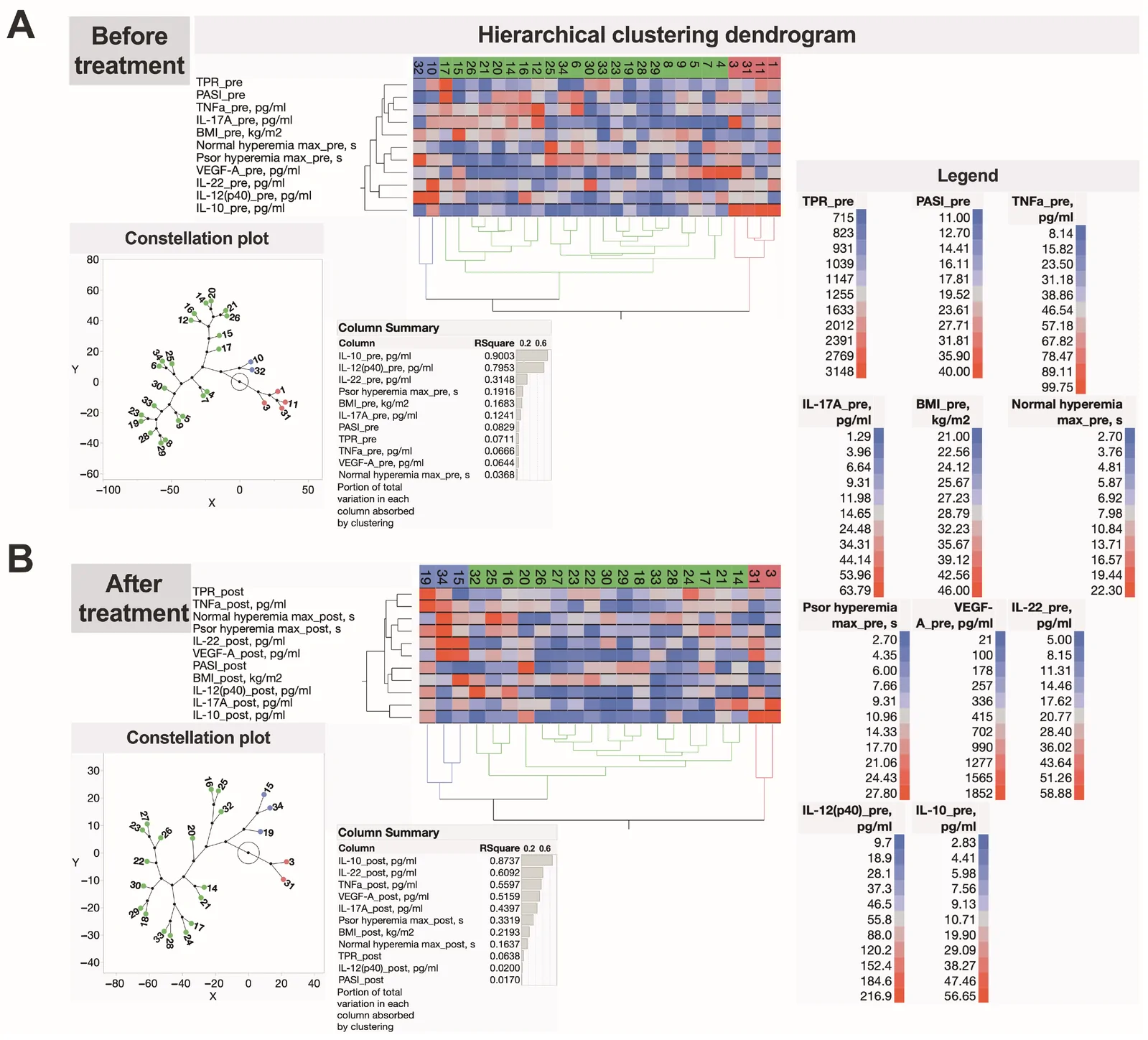
Cluster analysis of studied factors in psoriasis patients before (**A**) and after (**B**) treatment, presented as a Ward’s hierarchical clustering dendrogram with constellation plot (colour dots reflect respective cluster).

The pre-treatment clustering solution separated patients into two major clusters, characterised by a low-expression and high-expression phenotype across the measured variables, as visible in the heatmap colour distribution (Figure 12A). The variable contributing most strongly to cluster separation, as quantified by the proportion of variance absorbed by clustering (R^2^), was IL-10 (R^2^ = 0.90), followed by IL-12(p40) (R^2^ = 0.80) and IL-22 (R^2^ = 0.31). The remaining variables—including PASI, TPR, TNF-α, VEGF-A, IL-17A, and both LDF indices—contributed relatively little to pre-treatment cluster structure (R^2^ ≤ 0.17 for all). These data suggest that the pre-treatment clustering architecture may be predominantly driven by the IL-10/IL-12(p40) immunological axis, generating the hypothesis that inter-patient heterogeneity in psoriasis at baseline is structured primarily around the balance between anti-inflammatory (IL-10) and Th1-polarising (IL-12(p40)) mediators rather than around clinical severity or vascular indices per se. A small subgroup of 2–3 patients (including patients 11 and 31) appeared spatially separated from the main cluster in the constellation plot, potentially representing atypical immunological phenotypes within the cohort; however, the clinical or biological significance of this separation cannot be established from the present data.

The post-treatment clustering solution also produced two main patient clusters, but with a markedly different set of clustering drivers (Figure 12B). IL-10 again dominated cluster separation (R^2^ = 0.87), but the contributions of IL-22 (R^2^ = 0.61), TNF-α (R^2^ = 0.56), and VEGF-A (R^2^ = 0.52) increased substantially relative to the pre-treatment solution. Conversely, IL-12(p40) dropped markedly in its clustering contribution (R^2^ = 0.02 post-treatment versus 0.80 pre-treatment). The post-treatment constellation plot showed a more dispersed patient arrangement relative to pre-treatment, with patient 19 appearing as a notable outlier.

These observations are consistent with the hypothesis that treatment differentially suppresses the Th1-polarising (IL-12(p40)) component of the pre-treatment immunological landscape while leaving inter-patient heterogeneity in pro-inflammatory (TNF-α, IL-22) and angiogenic (VEGF-A) mediators as the primary axes of residual variation post-treatment. However, this interpretation is exploratory: the clustering solutions presented here are derived from a single cohort without external validation, the two-cluster solution was selected on visual and variance-based criteria without formal stability testing, and alternative cluster structures cannot be excluded. Prospective validation in larger independently assembled cohorts with pre-specified clustering criteria is required before these patterns can inform patient stratification or clinical decision-making.

## 4. Discussion

This study provides an integrated evaluation of inflammatory cytokines, angiogenic mediators, systemic vascular resistance, and microvascular reactivity in patients with chronic plaque psoriasis before and after 12 months of therapy. The findings demonstrate that clinical improvement, reflected by a marked reduction in PASI, is accompanied by coordinated immunological and vascular changes. These findings reinforce the concept of psoriasis as a systemic inflammatory disease rather than a purely cutaneous disorder and highlight the potential dissociation between cutaneous improvement and vascular recovery.

### 4.1. PASI as a Sensitive Indicator of Therapeutic Response

The substantial decline in PASI following treatment confirms the clinical effectiveness of the therapeutic regimen. PASI demonstrated excellent discriminatory performance between pre- and post-treatment states (AUC = 0.9692, *p* < 0.0001). This reinforces PASI as a robust clinical endpoint and validates its use as a reference measure for interpreting biomarker dynamics [11].

### 4.2. Cytokine Dynamics Reflect Modulation of the IL-23/Th17 Axis

IL-17A, a central effector cytokine in the IL-23/Th17 pathway, decreased significantly after therapy (*p* = 0.0067) despite modest ROC performance (AUC = 0.5800). This pattern suggests that IL-17A is responsive to treatment but exhibits interindividual variability, possibly reflecting differences in disease chronicity, metabolic status, or treatment mechanism [23].

IL-22 showed one of the most pronounced reductions, with strong discriminatory ability (AUC = 0.7833, *p* < 0.0001) and improvement in 79% of patients. Given IL-22’s role in keratinocyte proliferation and epidermal barrier disruption, its decline aligns with clinical improvement and supports its utility as a biomarker of therapeutic response [24].

IL-12(p40), shared by IL-12 and IL-23, also decreased significantly (*p* = 0.0002) and showed moderate ROC performance (AUC = 0.6894). Its strong association with systemic vascular resistance (χ^2^ = 9.38, *p* = 0.0022) suggests that IL 12(p40) may link inflammatory and hemodynamic pathways [13].

TNF-α remained strongly correlated with PASI (r = 0.536, *p* = 0.0013), consistent with its suggested role in psoriasis pathogenesis. Its persistent association with disease severity even after therapy underscores its centrality in the inflammatory network [2,25].

IL-10, an anti-inflammatory cytokine, correlated positively with IL-17A both before (r = 0.448, *p* = 0.0078) and after therapy (r = 0.5806, *p* = 0.0003). This suggests a compensatory regulatory response to Th17-driven inflammation. The negative trend between IL-10 II and normal hyperemia max II (r = −0.390) may indicate that higher IL-10 levels accompany improved microvascular recovery [26].

### 4.3. Systemic Vascular Resistance and Microvascular Dysfunction in Psoriasis

A key finding of this study is that systemic vascular resistance and cutaneous microvascular reactivity did not fully normalise despite marked clinical improvement, highlighting a dissociation between skin clearance and vascular recovery with important implications for long-term cardiovascular risk management. Total peripheral resistance showed a non-significant trend toward reduction following therapy (*p* = 0.3468), with modest ROC performance (AUC = 0.6452, *p* = 0.0445), reflecting directional but incomplete hemodynamic normalisation at the group level and suggesting that vascular dysfunction may outlast visible inflammatory improvement through sustained endothelial activation, structural remodelling, or residual inflammatory signalling.

The chi-square analysis of cytokine–TPR associations (Figure 9) revealed a clinically informative shift between disease states. Before therapy, systemic vascular resistance was significantly associated only with IL-22 (χ^2^(1) = 26.02, *p* < 0.0001) and BMI (χ^2^(1) = 4.18, *p* = 0.0410), while IL-17A, TNF-α, VEGF-A, IL-10, and IL-12(p40) did not demonstrate significant pre-treatment associations. The positive IL-22–TPR relationship at baseline is consistent with its established role in endothelial activation and vascular permeability, suggesting that at peak inflammatory activity this cytokine contributes to hemodynamic dysregulation beyond its cutaneous effects.

After therapy, significant TPR associations expanded to five variables, reflecting a qualitative reorganisation of the inflammatory–vascular landscape rather than simple attenuation. TNF-α demonstrated the strongest and most clinically notable post-treatment association (R^2^ = 0.226, χ^2^(2) = 94.84, *p* < 0.0001), characterised by a pronounced exponential-like fitted curve in which TPR remained relatively stable at low-to-moderate TNF-α concentrations but increased sharply at the highest residual values. This non-linear threshold profile suggests that below a certain level of residual TNF-α-driven inflammation, vascular resistance approaches near-normal values, whereas above that threshold sustained pro-inflammatory signalling—through endothelin-1 upregulation, nitric oxide synthase inhibition, and reactive oxygen species-mediated vasoconstriction—perpetuates vascular tone dysregulation disproportionately. The emergence of TNF-α as the dominant post-treatment hemodynamic correlate is particularly noteworthy given that it did not reach FDR-corrected significance as a treatment-responsive biomarker (q = 0.0961), suggesting that its haemodynamic relevance is concentrated in the subset of patients with the highest residual post-treatment concentrations. Taken together with its strong pre-treatment correlation with PASI (r = 0.536, *p* = 0.0013), TNF-α demonstrates dual clinical relevance in psoriasis: as a disease severity marker in the active state and as a potential hemodynamic risk marker in the treated state.

BMI emerged as the strongest overall post-treatment TPR correlate in terms of model fit (R^2^ = 0.130, χ^2^(1) = 40.72, *p* < 0.0001), with a pronounced negative linear relationship, suggesting that after reduction in the dominant cytokine burden, metabolic status becomes a more prominent independent determinant of residual vascular resistance. This finding implies that metabolic optimisation—weight management, dyslipidaemia treatment, glycaemic control—may be an important complementary cardiovascular risk reduction strategy in patients who achieve good dermatological control. IL-22 retained a significant post-treatment association (χ^2^(1) = 21.83, *p* < 0.0001), consistent with incomplete systemic immunological remission despite cutaneous clearance. VEGF-A demonstrated a newly significant post-treatment association (χ^2^(1) = 9.38, *p* = 0.0022) that, alongside its correlation with psoriatic skin hyperemia (r = 0.442, *p* = 0.0088), suggests residual angiogenic activity contributes to both microvascular reactivity delay and systemic hemodynamic burden through overlapping mechanisms. IL-17A and IL-12(p40) were not significant post-treatment, consistent with their preferential suppression by the biologic therapies predominant in this cohort.

Laser Doppler Flowmetry corroborated these systemic findings at the microvascular level: delayed time to peak hyperemia in psoriatic skin remained positively associated with TPR after therapy (r = 0.3447, *p* = 0.0459), indicating that local microvascular impairment and systemic hemodynamic burden share common pathophysiological determinants and that cutaneous vascular normalisation lags behind clinical remission. Collectively, these findings support a model in which the vascular consequences of psoriasis-associated inflammation are not simply attenuated by therapy but are qualitatively reorganised: residual TNF-α and metabolic factors, rather than the acute IL-22-dominated cytokine burden of active disease, become the primary drivers of vascular resistance in the treated state, and warrant monitoring alongside standard clinical endpoints to fully characterise long-term cardiovascular risk in psoriatic patients [8,27,28,29].

### 4.4. IL-12(p40) as a Potential Link Between Inflammation and Vascular Dysfunction

Among the cytokines studied, IL-12(p40) emerged as uniquely associated with systemic vascular resistance, demonstrating the strongest relationship with TPR in multivariate analyses. As a shared subunit of IL-12 and IL-23, IL-12(p40) occupies a central position at the interface of Th1- and Th17-driven immune responses.

The observed association between IL-12(p40) and vascular resistance suggests that this cytokine may act as a functional link between immune activation and hemodynamic regulation. While the correlation between IL-12(p40) and psoriatic skin hyperemia did not reach conventional statistical significance, the consistency of these associations across vascular domains supports a biologically plausible role for IL-12(p40) in modulating vascular tone and endothelial responsiveness.

These findings align with the emerging concept that specific inflammatory mediators may differentially affect vascular function, and they raise the possibility that immunological remission does not uniformly translate into vascular normalisation.

### 4.5. Clinical and Translational Implications

The present findings have important clinical implications. While PASI remains an excellent measure of cutaneous disease activity, it may not fully capture the systemic vascular burden associated with psoriasis. Persistent abnormalities in vascular resistance and microvascular reactivity following effective therapy suggest that cardiovascular risk assessment should remain a priority even in clinically well-controlled patients.

Integrated evaluation of immunological and vascular parameters may therefore offer added value in the long-term management of psoriasis, enabling identification of patients with residual vascular risk despite apparent dermatological remission. Future studies should examine whether targeted anti-inflammatory therapies can differentially restore vascular function and whether microvascular markers can serve as predictors of cardiovascular outcomes in psoriasis.

### 4.6. Network Level Interactions Reveal a Highly Integrated Inflammatory–Vascular System

The Bayesian network and hierarchical clustering analyses presented here were undertaken as exploratory, hypothesis-generating investigations of the multivariate dependency structure linking inflammatory, angiogenic, vascular, and clinical variables in psoriasis before and after therapy. These analyses should not be interpreted as establishing causal relationships, biological mechanisms, or generalisable patient subtype classifications; all inferences remain speculative in the context of the present sample size and observational design, and require prospective experimental and clinical validation.

With this framing established, the exploratory observations generated by these analyses offer several directions for future investigation. The pre-treatment Bayesian network identified PASI as the most extensively connected conditional hub (6 of 10 active edges; graph sparsity = 0.818), which is consistent with the established role of clinical disease severity as a shared correlate of immunological activation, angiogenic signalling, and microvascular dysfunction in psoriasis. The conditional dependency between TPR and IL-12(p40) observed in the pre-treatment network is consistent with published evidence linking IL-12/23-driven Th1 polarisation to endothelial activation and vascular resistance, and may identify IL-12(p40) as a candidate mediator of the inflammatory–hemodynamic interface in psoriasis—a hypothesis that warrants investigation in mechanistic studies with appropriate causal designs.

The post-treatment network showed a reduction in active conditional dependencies (7 edges, sparsity = 0.873), with the emergence of an IL-10/IL-17A conditional association absent pre-treatment. This pattern may suggest that after therapeutic suppression of the dominant inflammatory cascade, a residual regulatory–effector axis becomes detectable within the conditional dependency structure—potentially reflecting an immunomodulatory reconfiguration rather than simple inflammatory suppression. However, this interpretation is highly speculative given the network sample size, and the observed structural change may equally reflect statistical artefact or the loss of suppressing confounders rather than a genuine biological reorganisation.

The hierarchical clustering findings suggest that inter-patient heterogeneity in psoriasis is structured differently before and after therapy: pre-treatment variation may be dominated by the IL-10/IL-12(p40) axis, while post-treatment residual heterogeneity appears more strongly associated with pro-inflammatory (TNF-α, IL-22) and angiogenic (VEGF-A) mediators. If replicated, this shift could have implications for understanding which immunological axes drive residual disease activity after treatment and which biomarkers are most informative for monitoring therapeutic completeness. The observation that IL-12(p40)’s clustering contribution dropped from R^2^ = 0.80 pre-treatment to R^2^ = 0.02 post-treatment, while VEGF-A’s contribution increased from R^2^ = 0.06 to 0.52, is particularly notable as a hypothesis-generating finding and is consistent with the concurrent FDR-confirmed treatment responsiveness of VEGF-A (q = 0.0132) identified in the primary paired comparisons.

Taken together, the network and clustering analyses offer a complementary, systems-level perspective on the inflammatory–vascular reorganisation accompanying psoriasis therapy, identify candidate mechanistic hypotheses for experimental follow-up, and reveal potential biomarker axes for residual patient heterogeneity after treatment. None of these observations should be interpreted as confirmatory of the biological pathways they appear to implicate. Their principal value lies in generating specific, testable hypotheses for prospective investigation in larger cohorts with pre-specified analytical designs and independent validation samples.

### 4.7. Integrated Interpretation

Taken together, the findings demonstrate:

- Strong clinical improvement (PASI reduction);
- Coordinated suppression of IL-17A, IL-22, and IL-12(p40);
- Persistent TNF-α association with disease severity;
- Angiogenic activity (VEGF-A) linked to IL-22 and microvascular delay;
- Systemic vascular resistance influenced by IL-12(p40);
- Microvascular dysfunction that improves but does not fully normalise;
- A reorganised but still interconnected inflammatory–vascular network after therapy.

These results support a model in which psoriasis is driven by a multi-layered interaction between Th17-mediated inflammation, angiogenesis, and systemic vascular dysfunction. Therapeutic improvement reduces inflammatory load but may not fully reverse vascular abnormalities, highlighting the importance of long-term monitoring of cardiovascular risk in psoriatic patients.

### 4.8. Strengths and Limitations

A major strength of this study is its longitudinal design and integrative approach, combining cytokine profiling, systemic hemodynamics, and microvascular assessment.

However, several limitations of the present study should be acknowledged when interpreting these findings.

First, treatment heterogeneity: psoriasis patients received clinically indicated systemic therapy according to individual clinical characteristics, resulting in a heterogeneous treatment portfolio that included both conventional systemic agents and biologic therapies. This real-world treatment setting was intentionally retained to enhance external validity, but it limits the ability to attribute immunological and vascular changes to any specific therapeutic mechanism. Therapy-specific analyses were not feasible within the current sample size.

Second, absence of cardiovascular risk factor adjustment: important cardiovascular covariates including hypertension, type 2 diabetes, dyslipidemia, smoking status, and concurrent cardiovascular medications (antihypertensives, statins) may independently influence vascular function and inflammatory cytokine levels. Although patients with severe uncontrolled cardiovascular disease were excluded per the study protocol, residual confounding by these factors cannot be fully excluded, and formal covariate adjustment was limited by the available sample size.

Third, limited sample size: the cohort of 34 psoriasis patients and 19 controls, while appropriate for a longitudinal study incorporating intensive multimodal vascular phenotyping, limits statistical power for subgroup analyses, correlation coefficient stability, and the detection of small-to-moderate effect sizes. Results from secondary and exploratory analyses should be interpreted accordingly.

Fourth, exploratory statistical design: the Bayesian network analysis and Ward’s hierarchical clustering were applied in an exploratory, hypothesis-generating framework. With a patient-to-variable ratio of approximately 3:1 in the network analyses, overfitting and model instability are acknowledged risks. These analyses generate candidate mechanistic associations that require prospective validation in larger, independently recruited cohorts.

Fifth, absence of mechanistic validation: no experimental validation of the observed associations was performed. The correlations and network relationships identified here are associational in nature; no in vitro, ex vivo, or animal model data were obtained, and the reported relationships cannot be taken as evidence of biological mechanisms or causal pathways.

Sixth, observational design: the prospective observational design precludes causal inference. All associations reported represent correlational relationships that may reflect shared confounders, reverse causation, or unmeasured mediating variables.

Seventh, absence of investigator blinding: investigators performing LDF and Fino-meter assessments were aware of participants’ disease status due to the visible nature of psoriatic lesions. Although vascular measurements were recorded prior to disclosure of cytokine results to minimise analytical bias, the absence of full assessor blinding represents a potential source of bias.

Eighth, no cardiovascular outcome data: no clinical cardiovascular endpoints were assessed in this study. The cardiovascular implications of the observed persistent vascular associations are therefore speculative and require prospective validation in adequately powered longitudinal outcome studies before clinical recommendations can be made.

## 5. Conclusions

This study provides an integrated longitudinal evaluation of immunological, angiogenic, hemodynamic, and microvascular parameters in patients with chronic plaque psoriasis before and after 12 months of therapy, combining conventional statistical approaches with multivariate network-based analyses to characterise the coordinated behaviour of the inflammatory–vascular system across treatment states.

Effective long-term therapy produced substantial clinical improvement, as reflected by a marked reduction in PASI (*p* < 0.0001, AUC = 0.9692), accompanied by statistically significant and FDR-confirmed reductions in four of the six cytokines measured. IL-22 and IL-12(p40) demonstrated the strongest and most consistent treatment responsiveness, reflecting effective modulation of the IL-23/Th17 axis, while VEGF-A and IL-17A also reached FDR-corrected significance (all q < 0.05). TNF-α and IL-10 did not achieve FDR-corrected significance, with TNF-α retaining a strong correlation with disease severity (r = 0.536, *p* = 0.0013) throughout the observation period, consistent with its central and persistent role in psoriatic inflammation.

Despite marked clinical improvement, systemic vascular resistance and cutaneous microvascular reactivity showed only partial normalisation, providing evidence of a dissociation between skin clearance and vascular recovery. The pattern of cytokine–TPR associations underwent a qualitative reorganisation following therapy: the pre-treatment landscape was dominated by IL-22 as the primary vascular resistance correlate, while the post-treatment state was characterised by a pronounced non-linear threshold relationship between TNF-α and TPR (R^2^ = 0.226, χ^2^(2) = 94.84, *p* < 0.0001) and by the emergence of BMI as the strongest overall hemodynamic correlate (R^2^ = 0.130, χ^2^(1) = 40.72, *p* < 0.0001). This shift suggests that residual pro-inflammatory signalling and metabolic burden, rather than the acute angiogenic cytokine activity that characterises active disease, become the dominant drivers of vascular resistance in the treated state. Persistent associations between VEGF-A, microvascular reactivity delay, and systemic vascular resistance further indicate that angiogenic activity continues to contribute to vascular dysfunction beyond the resolution of cutaneous disease.

Exploratory Bayesian network and hierarchical clustering analyses identified candidate structural patterns in the multivariate inflammatory–vascular dependency architecture that are consistent with therapy-associated simplification of conditional dependencies and a shift in the drivers of inter-patient heterogeneity from IL-12(p40) toward pro-inflammatory and angiogenic mediators after treatment. These findings are hypothesis-generating and require prospective validation in larger independently recruited cohorts before mechanistic or clinical conclusions can be drawn.

Collectively, the results reinforce the characterisation of psoriasis as a systemic inflammatory disease with sustained vascular involvement that persists beyond clinical remission. PASI, while an excellent indicator of cutaneous therapeutic response, does not fully capture the residual vascular burden in treated patients. Integrated monitoring of immunological markers—particularly TNF-α and VEGF-A—alongside metabolic parameters and microvascular indices may provide added value in characterising long-term cardiovascular risk in psoriatic patients who have achieved apparent dermatological control. Future studies should examine whether therapy-specific immunological remission profiles differentially predict vascular normalisation, and whether microvascular and hemodynamic markers can serve as early indicators of cardiovascular risk in this population.

## Figures and Tables

**Table 1:** The baseline characteristics. The data are expressed as a number (*n*), or mean (±SD).

	Control Group *n* = 19	Psoriasis Patients’ Group *n* = 34	*p* Value
Age, years	50 (11)	41 (12)	>0.05
Men/Women	16/3	28/6	>0.05
Body mass index (BMI), kg/m^2^	29 (5)	27 (5)	>0.05
Psoriasis area and severity index (PASI) before/after therapy	---	20.6 (7.7)/4.1 (5.3.)	<0.0001

**Table 2:** FDR-corrected results for six paired cytokine comparisons. Four cytokines confirmed as discoveries (Q = 5%); two interpreted as trend-level findings.

Rank	Cytokine	*p* (Uncorrected)	q (BH-FDR)	Significant?	Interpretation
1	IL-22	<0.0001	< 0.001	Yes	Discovery; most responsive cytokine
2	IL-12(p40)	0.0002	0.0006	Yes	Discovery; vascular coupling marker
3	IL-17A	0.0067	0.0132	Yes	Discovery; inter-individual variability noted
4	VEGF-A	0.0088	0.0132	Yes	Discovery; angiogenic modulation confirmed
5	TNF-α	0.0801	0.0961	No	Trend-level; PASI correlation preserved (r = 0.536)
6	IL-10	0.1023	0.1023	No	Trend-level; regulatory axis, not FDR-confirmed

## Data Availability

The data sets used and/or analysed during the current study are available from the corresponding author upon request.
